# Raman spectroscopy and multivariate analysis as potential tool to follow Alzheimer’s disease progression

**DOI:** 10.1007/s00216-022-04087-3

**Published:** 2022-05-19

**Authors:** Angela Gilda Carota, Beatrice Campanella, Renata Del Carratore, Paolo Bongioanni, Roberta Giannelli, Stefano Legnaioli

**Affiliations:** 1 Department of Chemistry and Industrial Chemistry, University of Pisa, Pisa, Italy; 2 Institute of Chemistry of Organometallic Compounds, ICCOM-CNR-Pisa, Pisa, Italy; 3 Institute of Clinical Physiology Research, IFC-CNR-Pisa, Pisa, Italy; 4 Spinal Cord Injuries Section, Azienda Ospedaliero-Universitaria, Pisa, Italy

**Keywords:** Raman spectroscopy, Dementia, Alzheimer’s disease, Multivariate analysis, Blood serum, Biomarkers

## Abstract

Raman spectroscopy is an emerging tool in the research and diagnosis of different diseases, including neurodegenerative disorders. In this work, blood serum samples collected from healthy controls and dementia patients were analysed by Raman spectroscopy to develop a classification model for the diagnosis of dementia of Alzheimer’s type (DAT). Raman spectra were processed by means of multivariate tools for multivariate analysis. Lower concentration levels of carotenoids were detected in blood serum from patients, which allowed for a good discrimination with respect to controls, such as 93% of correct predictions on the test set with random forest. We also hypothesize that carotenoid levels might be informative about the severity and progression of the disease, since the intensity of carotenoid signals decreased from the early stage to more severe patients. These encouraging results suggest the possibility to use Raman spectroscopy for the analysis of alternative biofluids (e.g. saliva) and the unobtrusive diagnosis of other neurodegenerative disorders.

**Graphical abstract:**

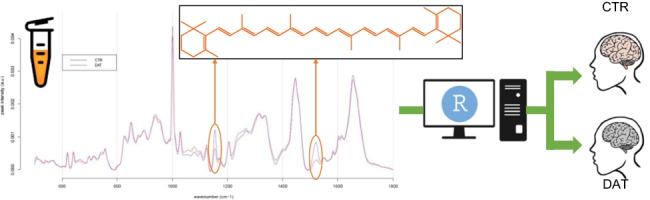

**Supplementary Information:**

The online version contains supplementary material available at 10.1007/s00216-022-04087-3.

## Introduction

Neurodegenerative diseases represent a growing cause of disability in industrialized countries. The extension of lifespan, due to improvements in public health, increased the incidences of age-related disorders, mostly of Alzheimer’s (AD) and Parkinson’s disease [1]. Dementia of Alzheimer’s type (DAT) is a severe neurodegenerative disorder of the brain characterized by loss of memory and cognitive decline [2]. Currently, neurodegenerative disease diagnosis is based on clinical symptoms, through a combination of psychiatric questionnaires and biomedical imaging methods, such as computerized tomography (CT), magnetic resonance imaging (MRI) and positron emission tomography (PET) [3, 4]. These methods are characterized by diagnostic accuracy but are slow, subjective and may not be predictive of the disease onset. Post-mortem pathological or molecular analyses of brain tissue (plaques and tangles) are then necessary for the verification of the pathology.

Biomarkers are essential to perform early diagnosis, monitor neurodegenerative disease progression, measure responses to therapies and stratify neurodegenerative disorders into their different subtypes. An ideal biomarker would distinguish DAT from other types of dementia. This is important, because treatment for these diseases might differ substantially [5]. Therefore, the research is pushing for the finding of reliable biomarkers for neurodegenerative diseases by novel techniques, and a wide range of molecular markers is under investigation in tissues and biofluids as well as through imaging. Mass spectrometry [6] and enzyme-linked immunosorbent assay (ELISA) [7] are the most developed and used techniques for the identification and quantification of biomarkers. Unfortunately, these techniques are slow and expensive. Raman spectroscopy is a label-free technique that rapidly provides chemical and structural information by detection of Raman scattering, i.e. inelastic collision of photons from molecules [8]. With respect to traditional biological assays, almost no sample preparation is required as well as no chemicals are necessary for the analysis, whose execution is also timesaving. The potential of the technique relies on its spectral fingerprint features, able to determine the presence of morphological–chemical alterations, due to a pathological condition, by slight changes in spectral profiles [9]. To highlight these spectral differences and to extract important biological information, the coupling with multivariate statistical methods is mandatory. To build reliable models, reference spectra recorded from tissue and cells with a known pathological status are used for the training of classification algorithms [10, 11]. This approach makes Raman a powerful, fast and sensitive tool for the analysis of biological samples.

Raman spectroscopy and its derivatives, coupled with multivariate analysis, have been applied mainly to cancer diagnosis [12–19] on a range of sample formats, including fixed cells and tissues, as well as non-invasive biofluid measurements [20] has been used also in diagnosis and study of other diseases due to viral or bacterial infections [21], even to study biofluid profile after COVID-19 infection [22], until it has been used in neurodegenerative disease diagnosis [23]. Raman spectroscopy has been also applied to study stem cells [24], cell lines in vitro [25] and metabolomics [26].

Oxidative stress, a pathophysiological mechanism in aging as well as in the cognitive impairment, can be markedly influenced by nutrition. Carotenoids are plant pigments responsible for bright red, yellow and orange hues in many fruits and vegetables that exhibit strong antioxidant properties. They have been associated with a reduced risk of several chronic diseases, such as cancers, diabetes, cardiovascular diseases and recent epidemiological studies that strongly suggest that consumption of carotenoid-rich foods reduces the incidence of some diseases including neurodegenerative diseases [27]. Carotenoids exert brain and cognitive protection, against onset and progression of Alzheimer, and serum levels of carotenoids seems to be positively associated with better cognition in aging subjects [28]. Nevertheless carotenoid-mediated health benefits are still limited, as the fundamental mechanisms of action in relation to human relevance are still not completely understood [29].

In the present work, we used Raman spectroscopy to find reliable biomarkers in blood serum samples with the aim of developing a fast and low-cost preliminary diagnostic method for DAT. We focused on blood serum carotenoids, since it is a less invasive and easy available biofluid respect to cerebral spinal fluid (CSF) [30].

## Materials and methods

### Human subject and clinical plasma sample collection

Blood plasma samples were collected from a total of 57 female subjects (considering the female prevalence in epidemiology of DAT; mean age ± SD, 78.8 ± 6.6). Twenty-six healthy controls (CTR) and 31 DAT patients from the Azienda Ospedaliero-Universitaria Pisana (AOUP) hospital were recruited. Patients underwent diachronically clinical evaluation and blood withdrawn for several years during the disease course of the disease. The study was conducted following the Declaration of Helsinki criteria and the Guidelines for Good Clinical Practice of the European Medicines Agency. The exclusion criteria for DAT patients were who suffered from any other neurological diseases, severe brain injuries and/or severe non-neurological illnesses. All clinical and biochemical data of patients are available upon practical request and verification of all ethical regulations.

DAT patients were diagnosed according to NIND criteria [31] and their disease severity scored according to the Clinical Dementia Rating (CDR) [32] at the time of collection. CDR is a 3-level assigned value: 1 indicates a mild degree of severity, and 2 refers to an intermediate stage, while 3 is assigned to the most severe stage of the disease. This parameter was assigned following clinical evaluation, neuropsychological and psychophysiological assessments carried out periodically by the medical staff.

The blood was collected into EDTA-treated tubes. Serum was obtained by centrifugation at 150 g for 15 min at room temperature, followed by centrifugation at 9,600 g and then stored at − 80 °C. All plasma aliquots underwent a single freeze–thaw cycle only.

### Raman spectroscopy

Blood serum samples were analysed by a Renishaw inVia confocal micro-Raman system, coupled with an optical Leica DLML microscope, equipped with a NPLAN objective 50 × with a numerical aperture of 0.75. The laser source used was a diode laser at 785 nm. The spectrometer consists of a single grating monochromator (1200 lines mm^−1^), coupled with a CCD detector, a RenCam 578 × 400 pixels (22 µm × 22 µm) cooled by a Peltier element. Spectral resolution of the spectrometer is 2.0 cm^−1^. Spectral calibration of the instrument was performed on the 520.0 cm^−1^ band of a pure silicon crystal.

Raman analysis was performed depositing 1 µL of blood serum on a microscope slide covered with an aluminium foil. The air-dried sample drop was irradiated by the 785 nm laser source with laser power on the sample of 41 mW. Spectra were acquired after 5 accumulations lasting 10 s each. Five spectra for each sample were collected to capture a possible inhomogeneity present in the dried drop. Spectra were sampled at the edge of the drop, taking into account the “coffee-ring effect”.

### Statistical analysis

During this work, different multivariate analysis methods were applied. Besides the principal component analysis (PCA), used to compute the dimensionality reduction of the dataset, discrimination methods such as principal component linear discriminant analysis (PC-LDA), principal component quadratic discriminant analysis (PC-QDA), partial least squares discriminant analysis (PLS-DA) and orthogonal partial least squares discriminant analysis (OPLS-DA), and a classification method, i.e. random forest (RF), were compared on the basis of their results in correct predictions of the test set.

Data analysis was performed using R software, both for spectra pre-treatment (*speaq* and *ChemoSpec* packages) and for multivariate analysis (*ChemoSpec*, *ropls*, *caret* and *randomForest* packages). Chemometric Agile Tool (CAT) software was used only to compute PC-LDA and PC-QDA analyses. During pre-treatment, spectra were normalized by total intensity, baseline corrected using *modpolyfit* (modified polynomial fitting) method and aligned and smoothed (Savitzky-Golay, window of 7 points, third polynomial filter). The analysed spectral range was between 500 and 1800 cm^−1^.

All data were mean centred before PCA, PC-LDA and PC-QDA. To perform discrimination and classification methods, the dataset was randomly divided into two subsets: a train set and a test set. The train set contained the 70% of the dataset while the test set the 30% for PC-LDA and PC-QDA. In PLS-DA and OPLS-DA train and test set were 50/50, while in RF, they contained the 75% (train) and 25% (test) of the dataset. RF model was built using a forest with 500 trees and mtry value of 6.

## Results

**Fig. 1:**
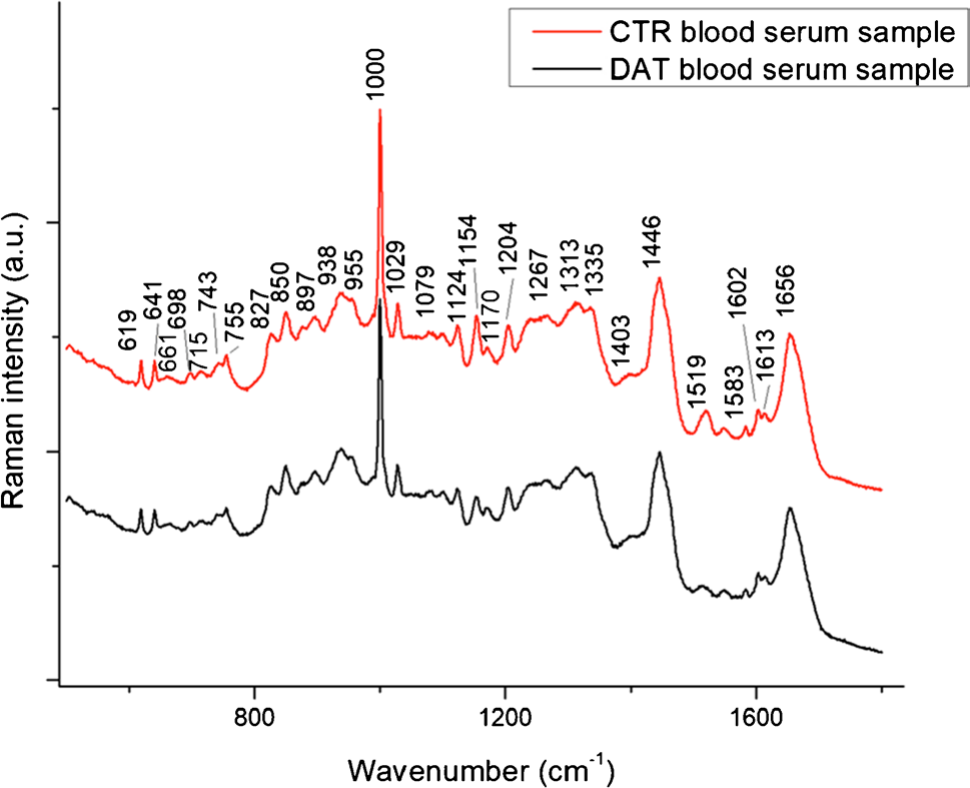
Raman spectra from CTR (red line) and DAT (black line) blood serum sample

Unprocessed Raman spectra of blood serum are reported in Fig. 1, and peak attribution is reported in Table 1 [33]. In supplementary information, Figure S1 showed spectral variances within and between CTR and DAT sample spectra, while Figure S2 showed the difference spectrum between the mean spectra of CTR and DAT. Most of the Raman peaks are related to the presence of proteins, e.g. the 1000 cm^−1^ phenylalanine peak and the 1656 cm^−1^ amide I band. Other substances are also present, such as carotenoids, phospholipids and haemoglobin (Hb). PCA was computed after spectra normalization and baseline correction to verify the homogeneity of each deposition, finding a low intra-sample variance (Figure S3, supplementary information).

**Table 1:** Blood serum Raman peaks vibrational modes

$\stackrel{\sim }{\nu }$(CM^−1^)	Assignment
619	Phenylalanine
641	Tyrosine
715	Polysaccharides
743	Phospholipids
755	Proteins; haemoglobin
827	Glutathione
850	Tyrosine
877	Tryptophan
897	C–O–C stretching
938	C–C stretching: α-helix
955	CH_2_ rocking
1000	Phenylalanine; carotenoids
1079	Phospholipids, O–P–O and C–C stretching
1124	Proteins; C–C phospholipids stretching
1154	Carotenoids (C–C)
1170	Tryptophan, phenylalanine; haemoglobin
1204	Tryptophan
1230–1282	Amide III
1300–1345	Tryptophan; α-helix; phospholipids
1403	Glutathione
1446	Phospholipids, CH scissoring in CH_2_
1519	Carotenoids (C = C)
1548	Tryptophan
1583	Proteins, tyrosine
1602	Tyrosine, phenylalanine
1613	Tyrosine, tryptophan C = C stretching
1656	Proteins, amide I α-helix; phospholipids

**Fig. 2:**
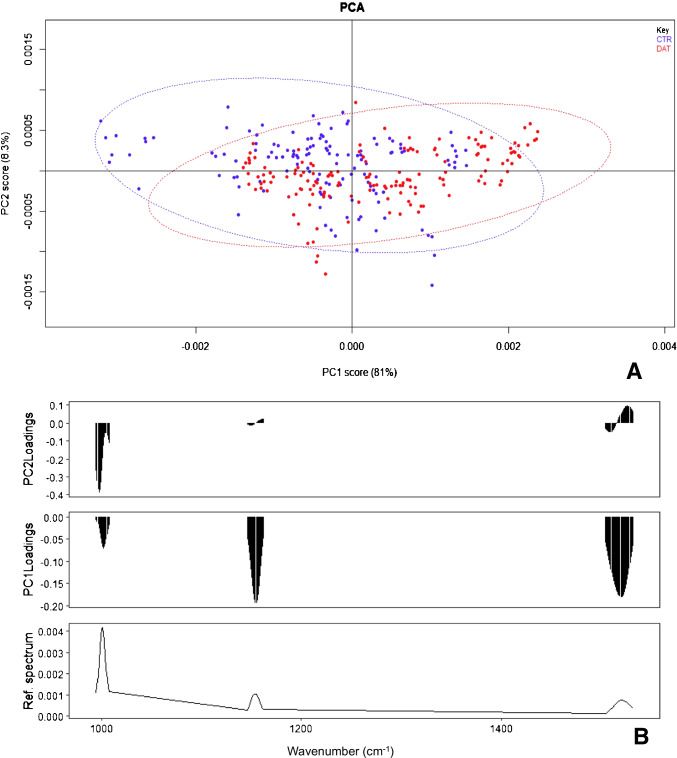
PC analysis: score plot (**A**) and loading plot (**B**) of the reduced spectrum dataset (carotenoids) (89.3% of variance explained on the two first principal components, 99.8% of variance explained by the first 10 PCs). CTR are in blue, while DAT in red

Multivariate analysis was applied to a total of 284 spectra (129 CTR, 155 DAT). Different discrimination and classification methods, computed on distinct datasets, were compared. In particular, data analysis was applied to the complete spectrum and to reduced spectra, containing only Hb (spectral ranges selected, 736–746 cm^−1^, 994–1008 cm^−1^, 1075–1085 cm^−1^, 1118–1129 cm^−1^, 1145–1162 cm^−1^, 1300–1345 cm^−1^, 1424–1470 cm^−1^, 1633–1685 cm^−1^) or carotenoid signals (spectral ranges selected, 994–1008 cm^−1^, 1145–1163 cm^−1^, 1503–1532 cm^−1^). Raman spectra of reference standard of carotenoids and Hb were reported in Figure S4 (see supplementary information). Dataset dimensionality was reduced by PC analysis. The first 10 PCA scores were extracted and used to compute PC-LDA and PC-QDA analyses, since these discrimination methods require a higher number of observables with respect to the number of variables. PCA plots of carotenoid signals were shown in Fig. 2, while PCA plots of the entire spectrum and Hb signals were shown in supplementary information, Figure S5 and S6 respectively.

Multivariate analyses results are showed in Table 2, where the correct predictions on the test set for the different methods are reported.

**Table 2:** Multivariate analysis results on the test set for each spectral range

Dataset	Method	Correct test predictions
Complete spectrum	PC-LDA	80%
	PC-QDA	81.2%
	PLS-DA	82.2%
	OPLS-DA	82.2%
	RF	91.5%
Haemoglobin	PC-LDA	70.6%
	PC-QDA	74.1%
	PLS-DA	70.9%
	OPLS-DA	70.2%
	RF	83.1%
Carotenoids	PC-LDA	71.8%
	PC-QDA	76.5%
	PLS-DA	72.3%
	OPLS-DA	73.7%
	RF	93%

The loading plot of PC analysis computed on the entire spectral range (Figure S5B, supplementary information) showed that carotenoids (peaks at 1000, 1154 and 1519 cm^−1^) [34] are responsible for the separation in the score plot between CTR and DAT, with the highest content of carotenoids correlated to CTR samples. Discrimination methods provided good results, with over the 80% of correct predictions on the test set. The 91.5% of the test set samples were correctly classified by RF, with the 12.7% of OOB estimate of error rate.

Since DAT patients tend to suffer of anaemia [35], the eventual change in spectrum profile of serum due to variable levels of Hb was evaluated. A mild separation between CTR and DAT could be appreciated in the related score plot (Figure S6, supplementary information). Furthermore, DAT samples (in red) were distributed following a trend coherent with the information given by the loadings, i.e. higher levels of Hb at negative values of PC1 and positive values of PC2. Among the discrimination methods, the best performance in test prediction was achieved by the PC-QDA model (74.1%), while RF provided the 83.1% of correct test predictions. Out-of-bag (OOB) estimate of error rate was 20.2%.

Analyses were applied also to the dataset reduced to carotenoids signals. In the PCA score plot (Fig. 2A), separation between classes was more accentuated, as the first PC contained the 81% of explained variance. Samples with more intense carotenoid signals were plotted at negative values of PC1, as showed by the loading plot (Fig. 2B). The discrimination methods provided slightly worse results with respect to the analysis applied to the entire spectral range, still better than those obtained from the Hb reduced spectra analysis. RF, on the other hand, provided the best result among all the analyses tested, since the 93% of the test set items were correctly classified, with the 8.9% OOB estimate of error rate.

**Fig. 3:**
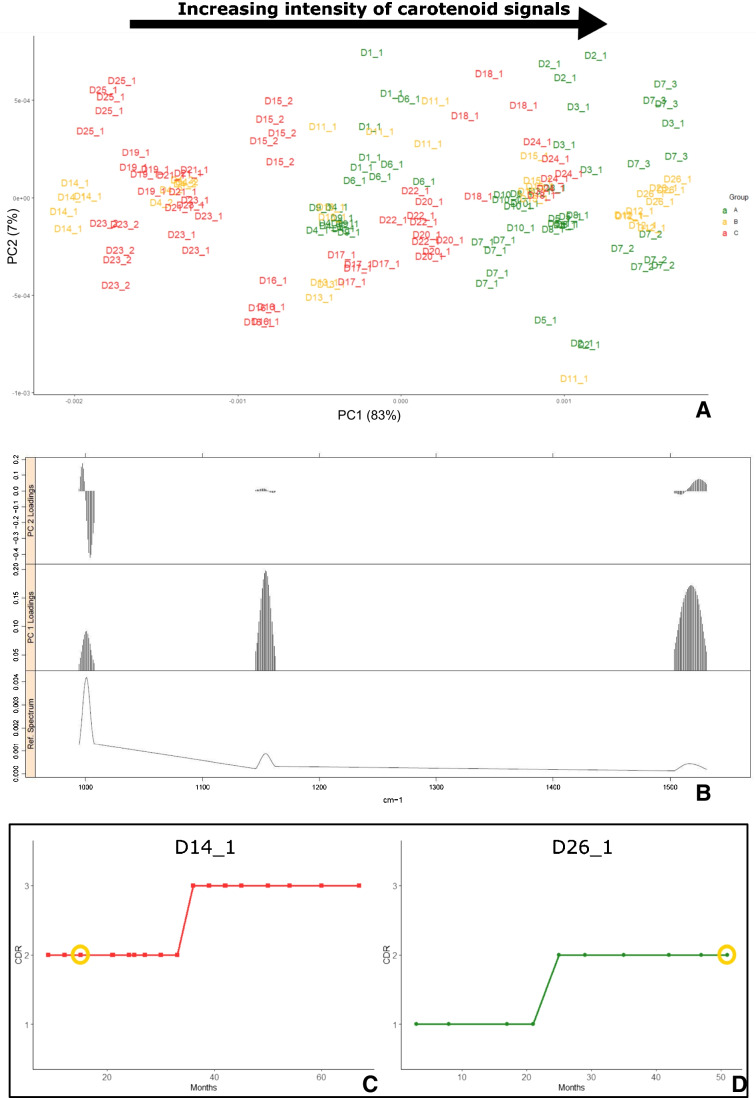
**A** PCA score plot of disease severity of DAT samples (99.8% of variance explained by the first 10 PCs). The arrow indicates the direction of increasing intensity of carotenoid peaks suggested by the loading plot, showed in **B**. In **C** and **D**, the CDR is plotted against the months of blood sample collection, for patients D14_1 and D26_1, respectively. The yellow circle indicates the date of collection of the analysed sample

PCA analysis was also computed on serum samples of DAT patients, classified in 3 classes of the clinical dementia rating (CDR) according to disease severity. PCA was applied to the carotenoid reduced dataset. Patients are coded as “Dx_y”, where “x” indicates the patient number, while “y” indicates the number of the sample collected from the same patient. According to the score plot (Fig. 3A), class *A* samples were at the right side of the plot, coherently with the loading plot, which collocated higher levels of carotenoids at positive values of PC1, while class *C* samples were on the left side. The intermediate stage patients, class *B*, corresponding to CDR = 2, had an ambiguous behaviour, since samples were apparently randomly dispersed around the plot. It could be helpful to correlate this data with disease progression, which, unlike severity, gives information about how quickly the disease advances. We found that samples whose disease had progressed quickly (i.e. in a few years they passed from mild to severe stage of severity) were plotted close to samples with CDR = 3, while patients with a slower and constant progression of the disease were plotted in proximity to those with mild degree of severity. As shown in Fig. 3C, samples from the subject renamed D14_1, which were plotted in the left side of the score plot, had a CDR = 2 when collected and since the onset discovery, but severity was going quickly to get worse to CDR = 3. Samples from the subject renamed D26_1 (Fig. 3D), instead, plotted on the right side, are related to a more stable period in CDR = 1, being collected after above 20 months after passage to CDR = 2. The score plot could give also some insights about patients follow-up. Indeed, in the analysed dataset, samples collected from the same patient at different disease stages are present. Disease worsening of the disease should be accompanied by a decrease in serum carotenoid levels that is a shift towards the left side of the plot. This trend was observed for samples D23_1 and D23_2, D4_1 and D4_2 and D15_1 and D15_2 (each couple were blood serum samples collected from the same patient in different times). The trend followed by samples D7_1, D7_2 and D7_3, instead, is apparently not attributable to disease progression. However, they remained on the right side of the plot, and they were collected from a patient who maintains a constant mild disease severity.

## Discussion

Using Raman spectroscopy as a tool for the monitoring of DAT progression is an attractive and challenging task. Strength points of the Raman technique are the short analysis time and the small amount of sample needed to carry out the analysis, the minimal sample preparation, the possibility to quickly pre-treat spectra prior to multivariate analysis and lower cost than other reported methods, as immunoselection techniques or mass spectrometry.

In this study, we investigated the spectral fingerprint of blood serum from DAT subjects compared to healthy controls. Statistical analysis was computed both on the entire spectral range (500–1,800 cm^−1^) and on two reduced spectral window.

Verma et al. studied by Raman spectroscopy the increase of Hb concentration in mice serum after haemolysis induced by sepsis due to a bacterial infection [36]. We looked for a similar variation in Raman spectra profile of serum, as DAT patients suffer from anaemia. However, we were not able to observe such variations in Hb peak intensity, as not enough Hb was released following haemolysis due to anaemia. It is worth noting that Verma et al. worked on mice serum and they induced haemolysis, while we worked on human collected samples. Variations of carotenoid levels, instead, were largely detectable, and they significantly influenced the separation among classes and prediction results. An important correlation by Raman spectroscopy between carotenoids and DAT is reported in this work. Since carotenoids are antioxidants, they could react with reactive oxygen species (ROS) originating from the formation of the Aβ plaques during the progression of the disease [37]. Thus, lower levels of carotenoids could be found in DAT serum samples. Carotenoid importance in the neurodegenerative disorder is confirmed by their use as therapeutics [38]. This class of compound is generally quantified by liquid chromatography, which requires sample preparation, the use of organic eluents and analysis time over 10 min [39]. On the other hand, identification by Raman is straightforward and proceeds without the need of additional chemical supplies. Moreover, although the statistical analysis is focused on carotenoids signals, the entire spectrum is acquired and then eventually available for multivariate analysis.

Summarizing, after testing different multivariate analysis methods, we could distinguish between serum samples of healthy controls and of DAT patients. In particular, very good results were obtained with random forest, both for the complete spectrum and for the reduced dataset containing the carotenoid peaks (both with correct test predictions above the 90%). Overall good results were obtained for the multivariate analysis of the complete spectrum, since all the methods achieved correct test prediction around 80%. In general, PC-QDA provided better results with respect to PC-LDA, while OPLS-DA was not always better than PLS-DA. Furthermore, each model was able to classify DAT samples with greater precision than CTR. However, despite the important role of carotenoids in the disease, discrimination methods reported better results for analyses conducted on the complete spectra rather than those conducted on the spectra reduced to the carotenoid peaks, as the other spectral features also influenced the construction of the model. Nevertheless, RF achieved the best analyses result processing this dataset, as it is robust to overfitting and it is considered more stable in the presence of very high dimensional parameter spaces that other machine learning algorithms [40]. The built models could be further improved introducing other methods that could reduce the dataset, e.g. genetic algorithms (GA), that, coupled to data analysis tools as PLS-DA, could improve the performance of the technique and also facilitate the identification of spectral regions that allow for better discrimination between classes.

Tau proteins and amyloid b peptide are generally investigated as biomarkers of AD. Very often, the study of these substances, however, involves the analysis of tissues or biofluids that require greater invasiveness [41, 42], or particular instrumentation or specially designed devices are used [43]. In the present work, we used Raman spectroscopy as a label-free method for the analysis of blood serum, which can be considered a minimally invasive biofluid and an easy sample to prepare. An important correlation was also found between carotenoids and disease severity and progression, as lower carotenoid levels characterized samples collected by patients affected by a higher degree of severity. Then, results suggested also that decreasing carotenoid levels might indicate the worsening of the disorder, as demonstrated by the trend of samples collected from the same subject at different stages of the disorder. It has been recently reported that low levels of circulating carotenoids could play a role in cognitive impairment, while higher blood concentrations of carotenoids are associated with lower risk of age-related cognitive dysfunction [44]. Therefore, circulating carotenoids could be considered informative of the state of cognitive function.

## Supplementary Information

Below is the link to the electronic supplementary material.

Supplementary file1 (DOCX 2.64 MB)
