# Supplementary material for: Raman spectroscopy and multivariate analysis as potential tool to follow Alzheimer’s disease progression
Source: Anal Bioanal Chem. 2022 May 19;414(16):4667–75. doi: 10.1007/s00216-022-04087-3 (PMC9117601; doi:10.1007/s00216-022-04087-3)
Supplement: Supplementary file 1 — Supplementary file1 (DOCX 2.64 MB) [file 216_2022_4087_MOESM1_ESM.docx]

Supplementary information to

Raman spectroscopy and multivariate analysis as potential tool to follow Alzheimer’s disease progression

Angela Gilda Carota^1,2^, Beatrice Campanella^2^, Renata Del Carratore^3^, Paolo Bongioanni^4^, Roberta Giannelli^3^_,_ Stefano Legnaioli^2*^

^1^ Department of Chemistry and Industrial Chemistry, University of Pisa, Pisa, Italy

^2^ Institute of Chemistry of Organometallic Compounds, ICCOM-CNR-Pisa, Pisa, Italy

^3^ Institute of Clinical Physiology Research, IFC-CNR-Pisa, Pisa, Italy

^4^Spinal Cord Injuries Section, Azienda Ospedaliero-Universitaria, Pisa, Italy

Corresponding author: stefano.legnaioli@cnr.it

1. **Supplementary figures**


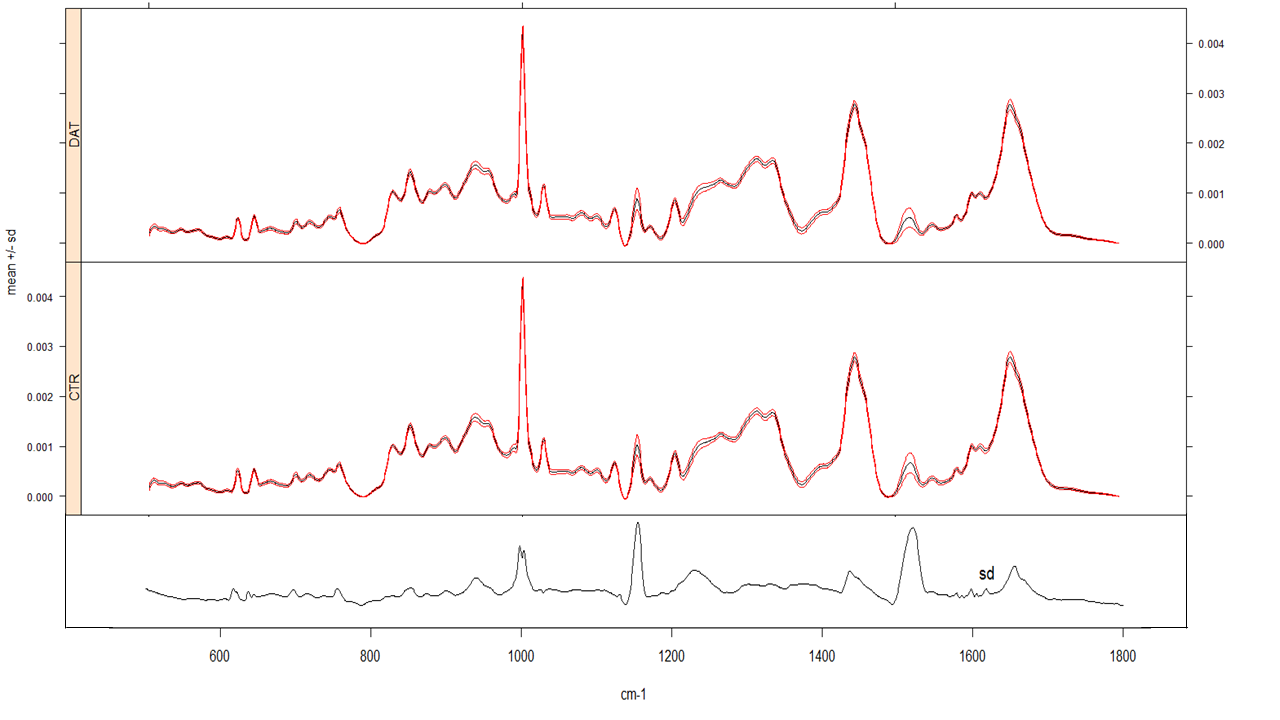


**A**

**B**

**C**

**Figure S1.** Spectral standard deviation (A, B) within the group (DAT and CTR respectively) and (C) between the groups. In (A) and (B) are showed the mean spectrum (in black) +/- the standard deviation (in red)


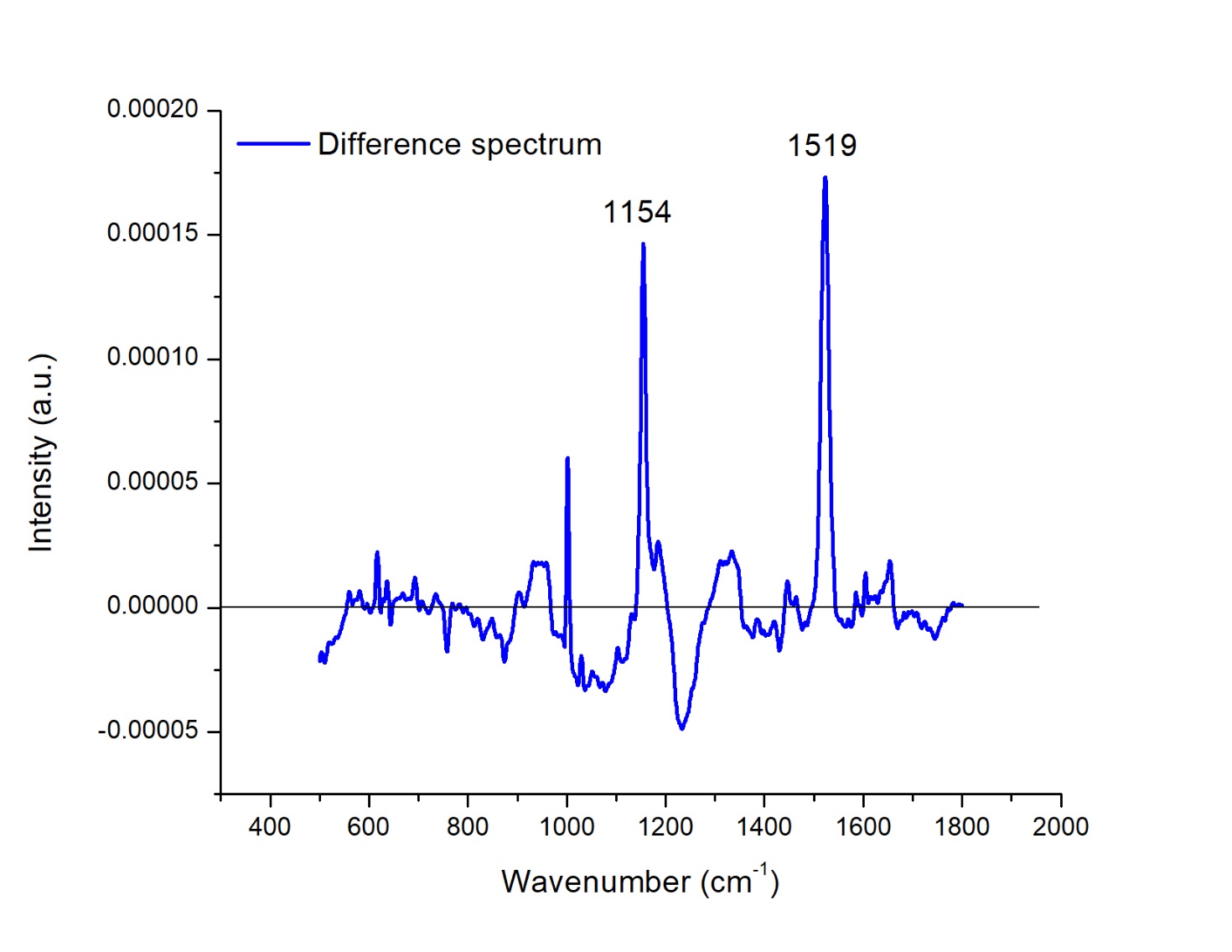


**Figure S2.** Difference spectrum between CTR and DAT mean spectra. A major difference is found among the carotenoids signals, at 1154 and 1519 cm^-1^.


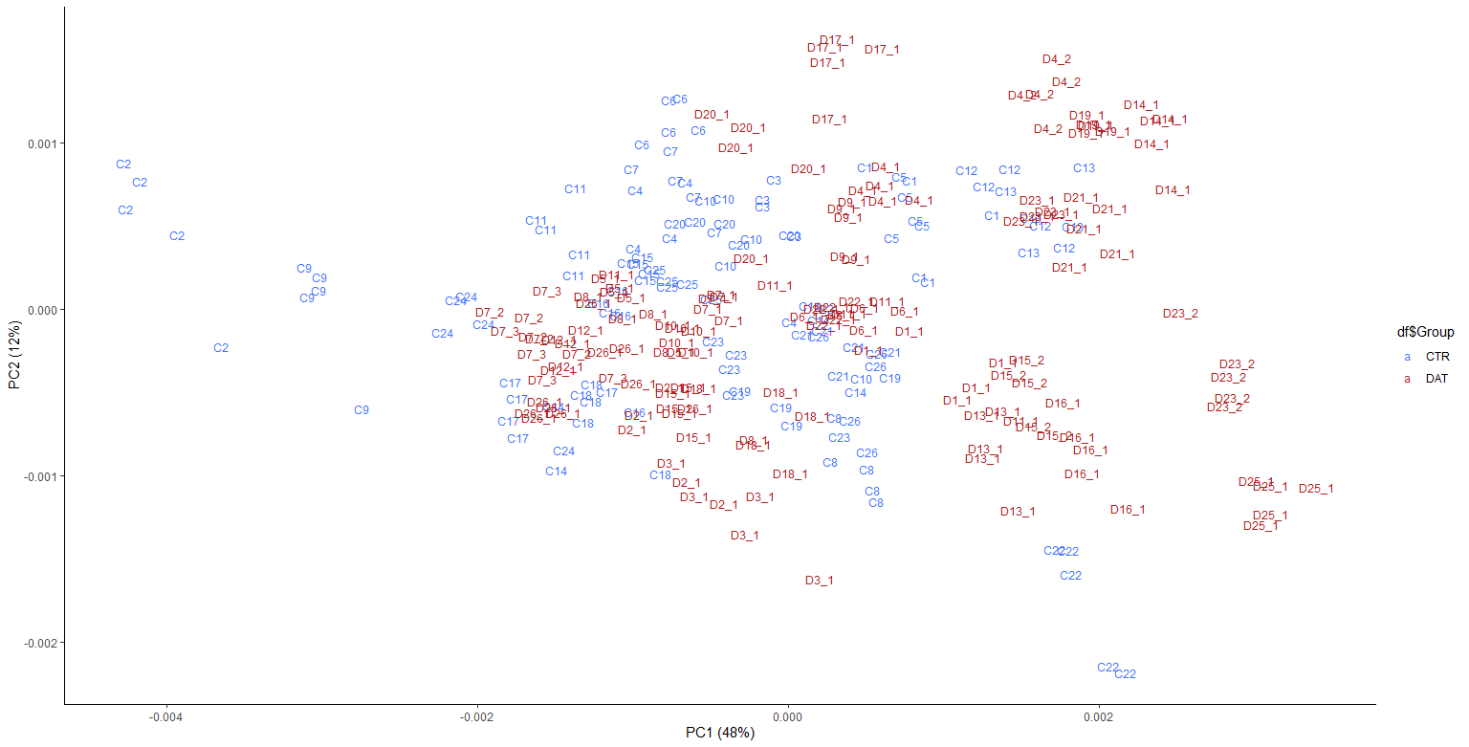


**Figure S3.** PCA score plot of the complete spectra dataset. The names of the samples are reported to evaluate intra-sample variance, as spectra acquired from the same sample are clustered. Controls are indicated as “Cx”, where “x” indicates the control number; patients are coded as “Dx_y”, where “x” indicates the patient number, while “y” indicates the number of the sample collected from the same patient.


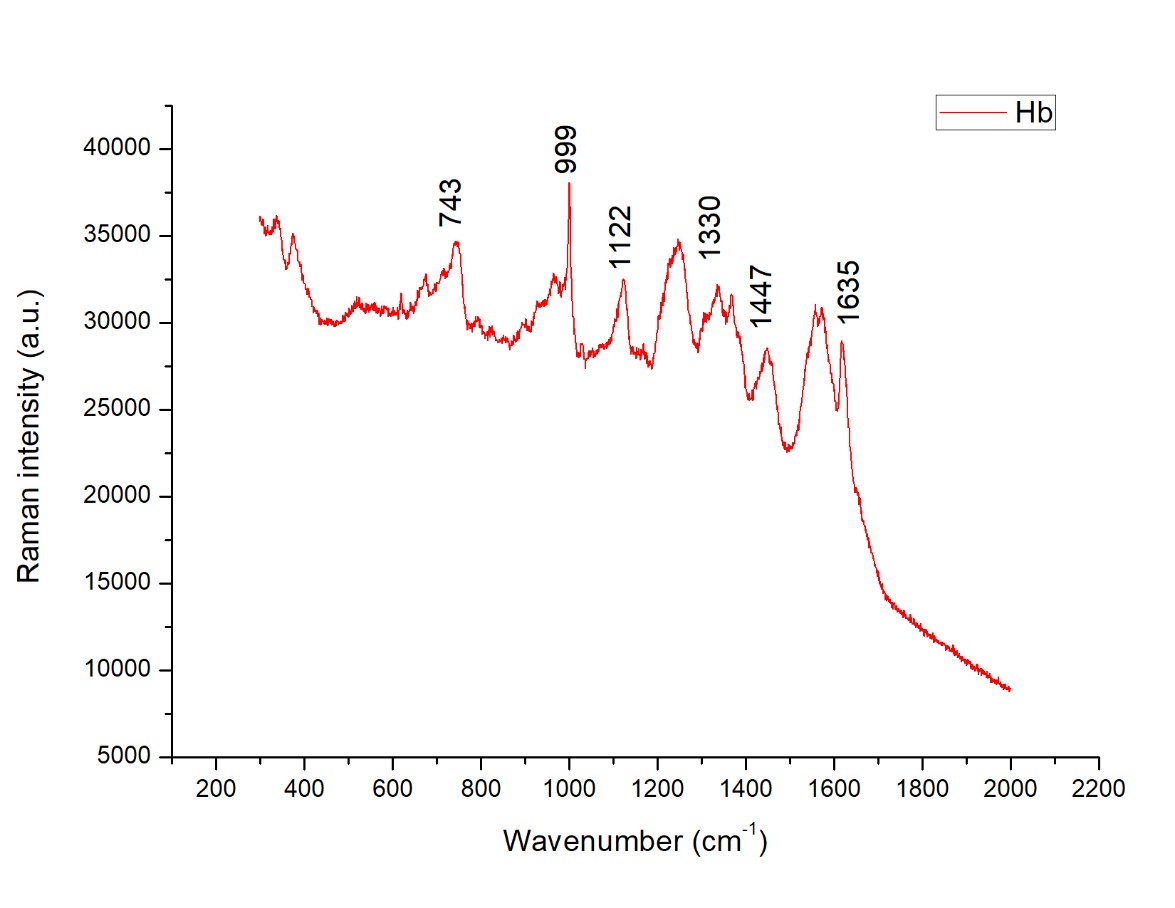

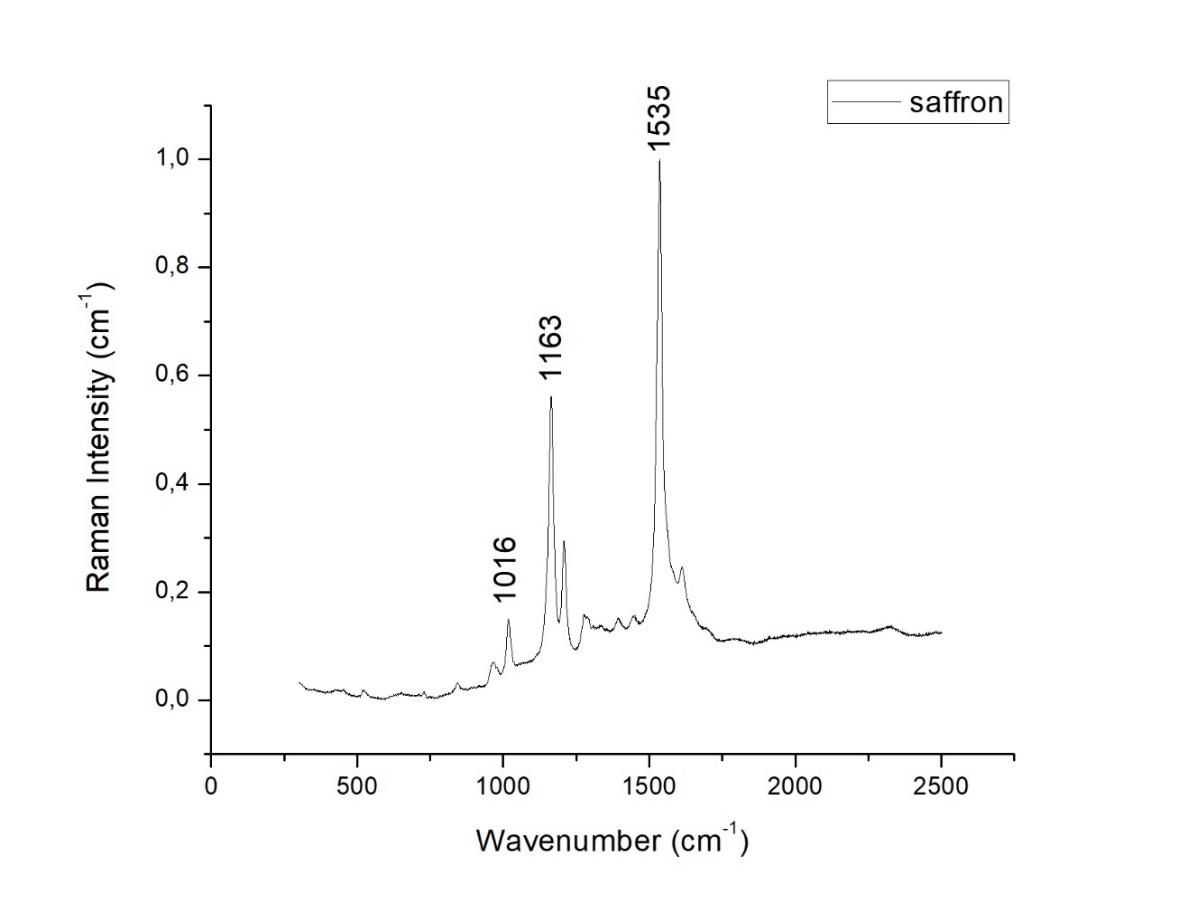


**Figure S4.** (A) Raman spectra of saffron standard sample. The main peaks of carotenoids (crocetin) are highlighted. (B) Raman spectra of haemoglobin standard sample. Spectra were acquired with a laser source of 785 nm on aluminium foil.


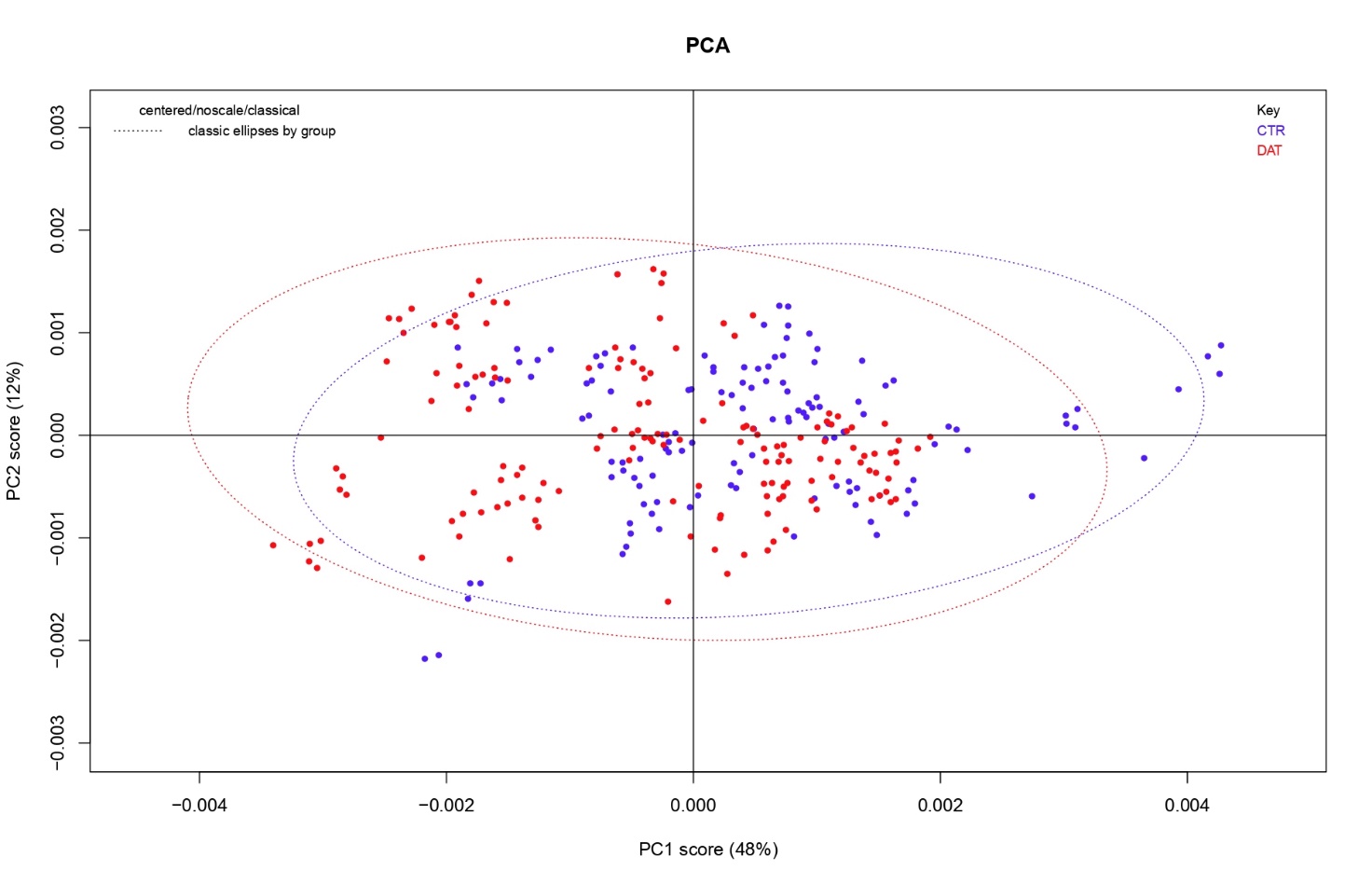


**A**


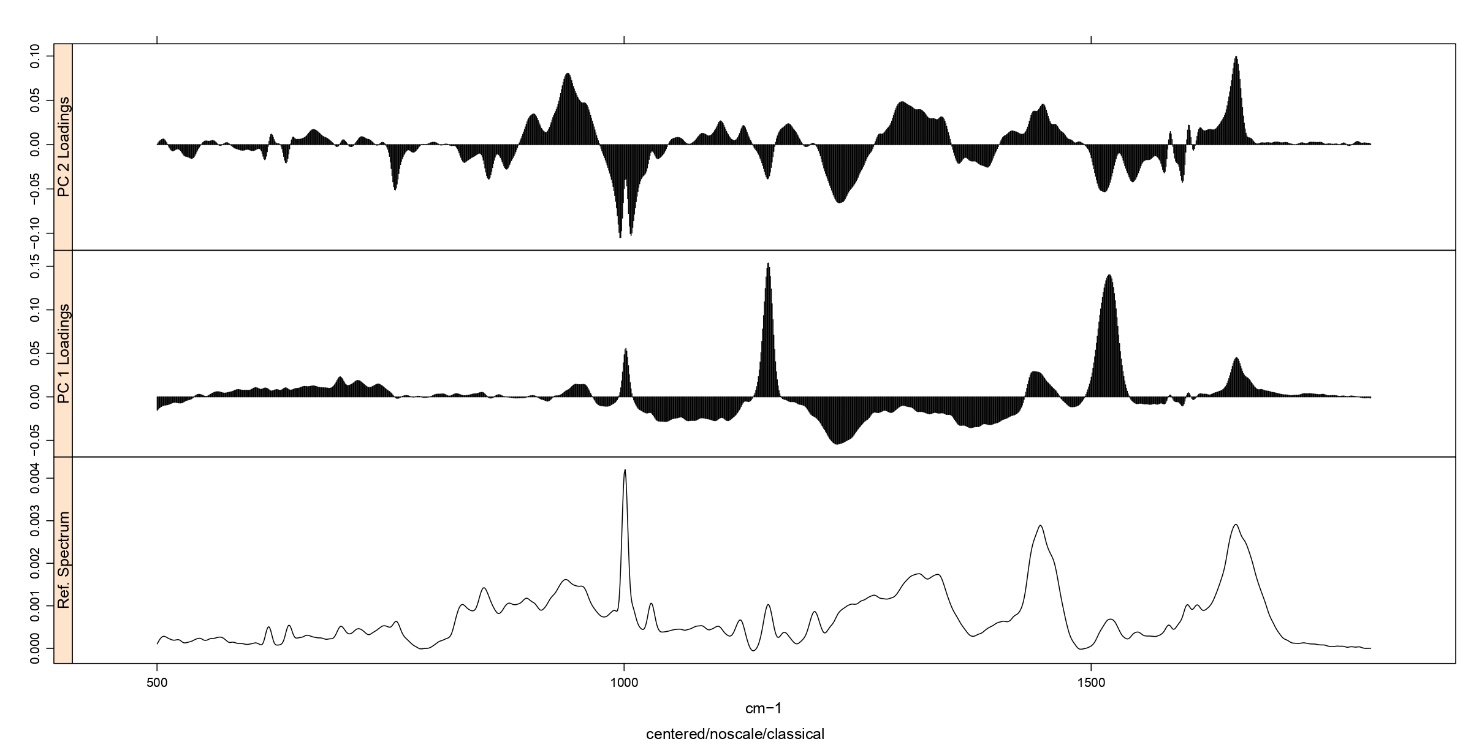


**B**

**Figure S5.** PCA score plot (A) and loading plot (B) of the complete spectrum dataset (60% of variance explained on the two first principal components, 90.7% of variance explained by the first 10 PCs). Controls (CTR) are in blue, while patients (DAT) in red.


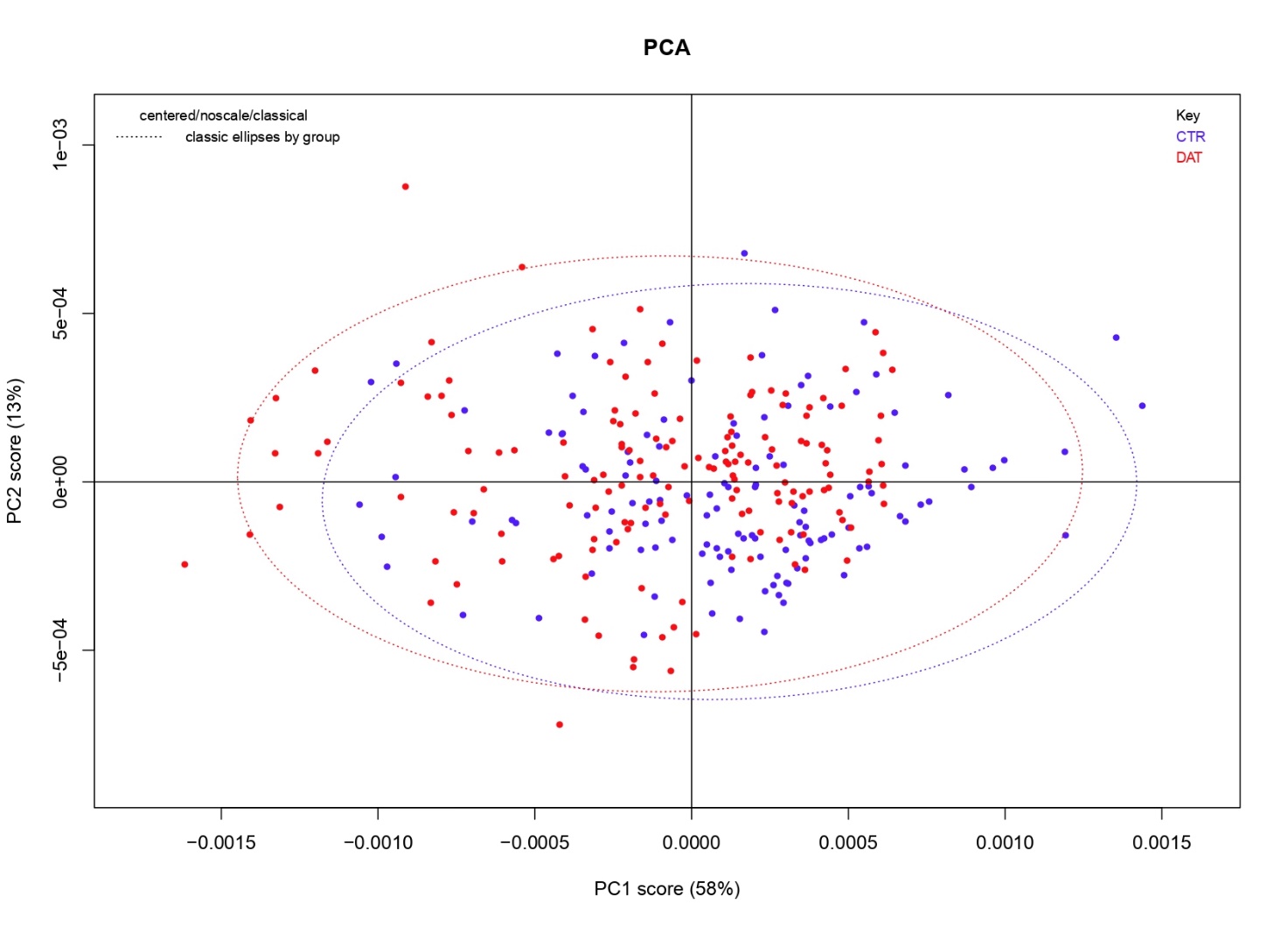


**A**


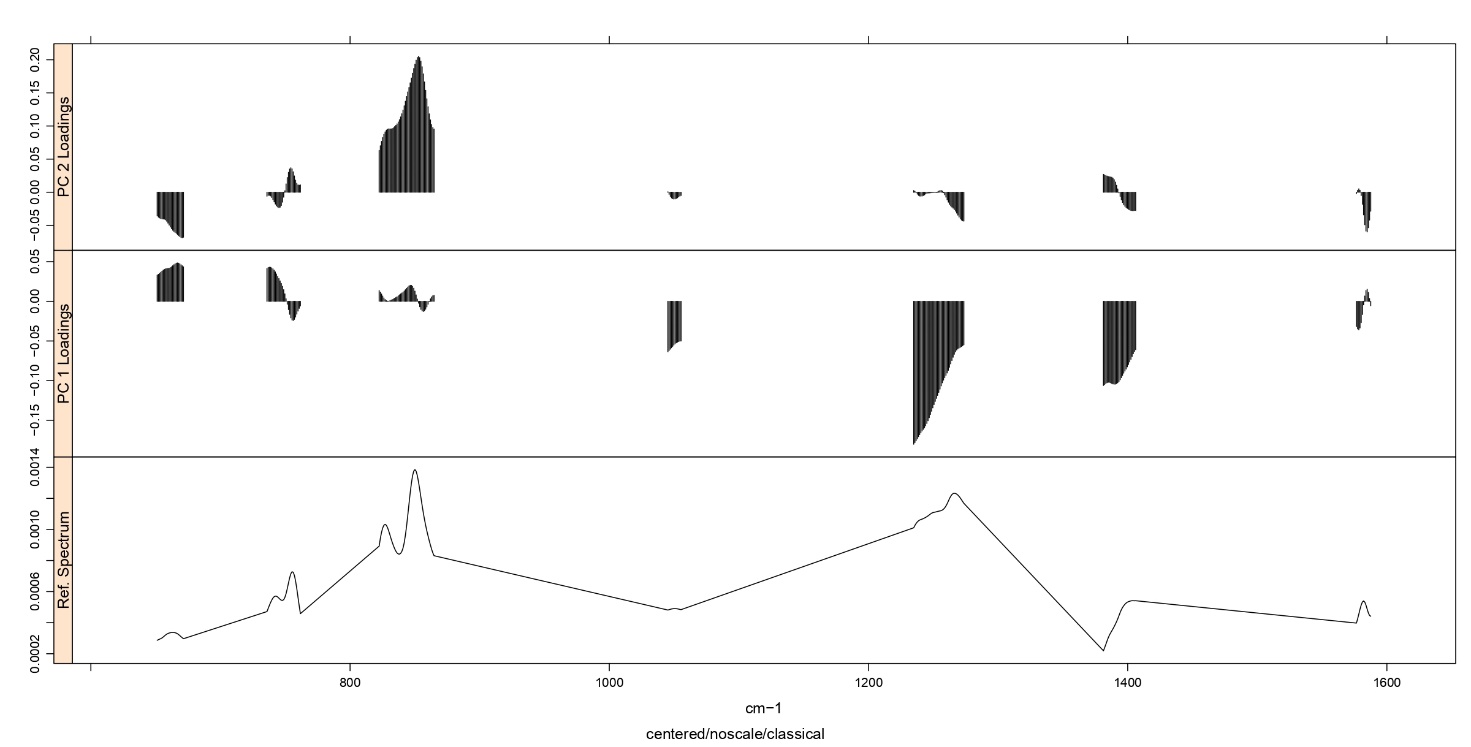


**B**

**Figure S6.** PCA score plot (A) and loading plot (B) of the reduced spectra dataset (Hb), (71% of variance explained on the two first principal components, 95.4% of variance explained by the first 10 PCs) of variance explained by the first 10 PCs). Controls (CTR) are in blue, while patients (DAT) in red.
